# Epstein–Barr virus (EBV) deletions as biomarkers of response to treatment of chronic active EBV

**DOI:** 10.1111/bjh.17790

**Published:** 2021-08-24

**Authors:** Cristina Venturini, Charlotte J. Houldcroft, Arina Lazareva, Fanny Wegner, Sofia Morfopoulou, Persis J. Amrolia, Zainab Golwala, Anupama Rao, Stephen D. Marks, Jacob Simmonds, Tetsushi Yoshikawa, Paul J. Farrell, Jeffrey I. Cohen, Austen J. Worth, Judith Breuer

**Affiliations:** ^1^ Institute of Child Health University College London London UK; ^2^ Department of Medicine University of Cambridge Cambridge UK; ^3^ Bone Marrow Transplantation Department Great Ormond Street Hospital for Children NHS Foundation Trust London UK; ^4^ Applied Microbiology Research Department of Biomedicine University of Basel Switzerland; ^5^ Clinical Bacteriology and Mycology University Hospital Basel Basel Switzerland; ^6^ Great Ormond Street Hospital for Children NHS Foundation Trust UK; ^7^ Department of Paediatric Nephrology Great Ormond Street Hospital for Children NHS Foundation Trust UK; ^8^ NIHR Great Ormond Street Hospital Biomedical Research Centre University College London Great Ormond Street Institute of Child Health London UK; ^9^ Department of Pediatrics Fujita Health University School of Medicine Toyoake Japan; ^10^ Section of Virology Department of Infectious Disease Imperial College Faculty of Medicine London UK; ^11^ Laboratory of Infectious Disease National Institute of Allergy and Infectious Diseases Bethesda MD USA; ^12^ Department of Immunology Great Ormond Street Hospital for Children NHS Foundation Trust London UK

**Keywords:** Epstein–Barr virus, chronic active EBV, defective viral genome

## Abstract

Chronic active Epstein–Barr virus (CAEBV) disease is a rare condition characterised by persistent EBV infection in previously healthy individuals. Defective EBV genomes were found in East Asian patients with CAEBV. In the present study, we sequenced 14 blood EBV samples from three UK patients with CAEBV, comparing the results with saliva CAEBV samples and other conditions. We observed EBV deletions in blood, some of which may disrupt viral replication, but not saliva in CAEBV. Deletions were lost overtime after successful treatment. These findings are compatible with CAEBV being associated with the evolution and persistence of EBV^+^ haematological clones that are lost on successful treatment.

## Introduction

Epstein–Barr virus (EBV) infects >95% of the population worldwide.^1^ A small number of patients develop life‐threatening persistence of high‐level EBV replication following an infectious mononucleosis syndrome, often associated with splenomegaly and hepatitis.^2^ Chronic active EBV (CAEBV) disease is characterised by infiltration of tissues by EBV^+^ T, natural killer (NK) or less frequently B cells and can progress into lymphoproliferative disease. Clonal expansion of EBV‐infected T or NK cells is well described.^3^

To date, CAEBV has been mostly described in Asian or South/Central American patients.^4^ Frequent deletions in the EBV genome have been found in samples from Japanese CAEBV patients (35%) and other EBV‐driven neoplasms.^5^, ^6^, ^7^ However, it is unclear if these deletions are specific for Asian EBV strains, whether they are present in viral genomes in different sites and how they evolve overtime.

In the present study, we sequenced EBV from serial blood samples obtained from three UK patients with CAEBV disease. The results were compared with sequences from saliva of patients with CAEBV and blood and tissue from other benign and malignant EBV‐related conditions.

## Methods

### Patients

**Fig 1:**
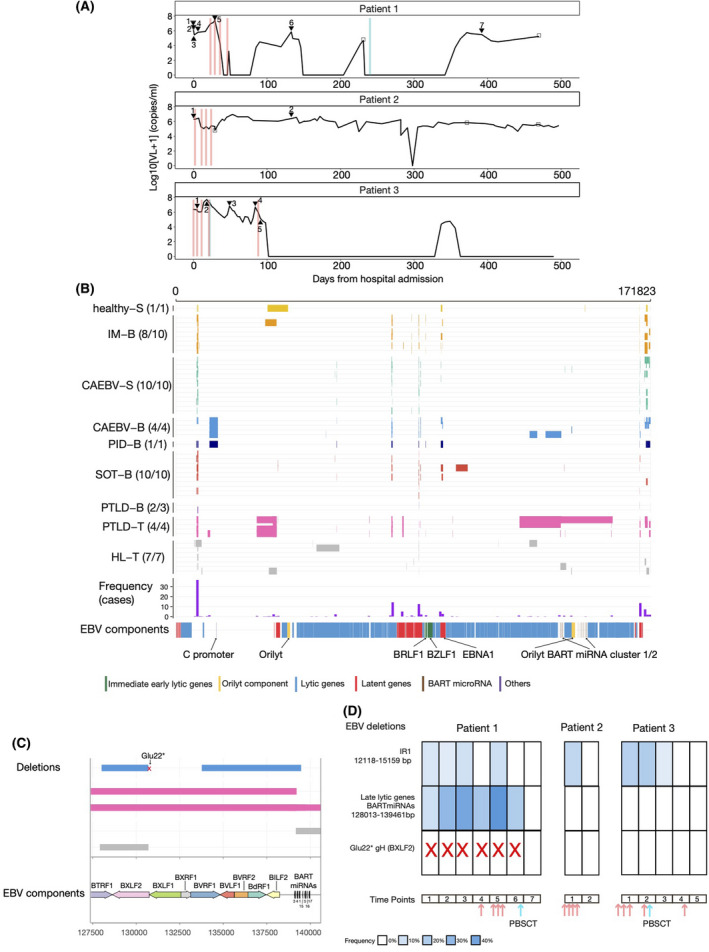
(A) Viraemia and treatment in a Great Ormond Street Hospital (GOSH) patient with chronic active Epstein–Barr virus (CAEBV). Black line represents viraemia over a period of 500 days after the first hospital record. Red lines: rituximab doses, blue lines: peripheral blood stem cell transplant (PBSCT), filled black triangle: deep‐sequencing samples, empty black squares: sequences samples that did not pass quality control. Patient 1 had a total of seven samples sequenced successfully of which four preceded starting treatment, one during rituximab treatment (time‐point 5), one after four doses of rituximab (time‐point 6) and the last one after PBSCT when the patient had EBV reactivation, but no symptoms (time‐point 7). Patient 2 had a deep‐sequencing sample taken before treatment and one after four doses of rituximab, to which they responded clinically despite the persistence of viraemia. Patient 3 had two previous episodes of EBV‐driven haemophagocytic lymphohistiocytosis (HLH) that spontaneously resolved. During the third episode, the patient started treatment with rituximab and two samples were collected during the first four doses (time‐point 1 and 2), two samples after PBSCT (time‐point 3 and 4) and a last sample after the last dose of rituximab (time‐point 5). The patient had EBV reactivation at low levels, with no sign of HLH. (B) Summary of deletions (≥30 bp) that were identified in EBV genomes. Each grey line represents an EBV genome from a single patient (if longitudinal samples from one patient were available, only one sample pre‐transplant and with the highest read depth was included). Only samples with deletions were visualised here. Colours indicate groups: yellow for salivary healthy samples (healthy‐S), orange for blood infectious mononucleosis (IM‐B), green for salivary CAEBV (CAEBV‐S), light blue for blood CAEBV (CAEBV‐B), dark blue for blood primary immunodeficiency (PID‐B), red for EBV‐positive solid organ transplant (SOT‐B), purple for blood post‐transfusion lymphoproliferative disease (PTLD‐B), pink for tumour PTLD (PTLD‐T) and grey for tumour from Hodgkin lymphoma (HL‐T). The purple histograms indicate the frequency of deletions (as number of samples) in each genomic region. The location of the main components of EBV genome are also shown based on GenBank sequence NC‐007605.1. (C) EBV components deleted in CAEBV and other malignancies. The horizontal lines represent deletions [using the same colour coded in (B)]. The location of EBV genes and components is shown based on NC‐007605.1. The number on top of each microRNA (miRNA) indicates its miRNA ID (three for example corresponds to ebv‐mir‐BART3). The red cross represents the nonsense variant (Glu22*, GAG→TAG at position 130 684 bp) identified in CAEBV samples in BXLF2 or glycoprotein H (gp85). (D) Summary of deleted components in longitudinal blood samples in patients with CAEBV. The *x*‐axis represents different time‐points of sampling [corresponding to number in (A)]. Both larger deletions (containing IR1 and late lytic genes and BART mRNAs) and the missense variant in BXLF2 are shown. The arrows indicate treatment, red for rituximab and blue for PBSCT. Transparency indicates the frequency (%) of viral genomes with the deletion. [Colour figure can be viewed at wileyonlinelibrary.com]

A total of 14 blood samples from three patients with CAEBV from Great Ormond Street Hospital (GOSH) were sequenced. The patients presented with a high EBV viral load (Fig 1A) and EBV‐driven haemophagocytic lymphohistiocytosis (HLH). Primary and secondary immunodeficiency was excluded and a diagnosis of CAEBV was considered (Data S1). All patients were treated with rituximab and followed the HLH‐94 protocol. As no response to treatment was seen for patients 1 and 3, EBV cell subsets was done and revealed EBV in T cells rather than B cells. They both underwent peripheral blood stem cell transplants (PBSCTs). Despite ongoing EBV viraemia, patient 2 did not receive a transplant because no further flares of HLH were observed after treatment. Viraemia lasted for >3 years and subsequent blood test revealed EBV in both B and T cells.

### Study design

The data were compared with 67 EBV sequences from asymptomatic (saliva, healthy‐S), infectious mononucleosis (blood, IM‐B), and other patients with CAEBV [a blood and a saliva sample from a CAEBV patient from GOSH and saliva samples from a paediatric USA CAEBV cohort, referred further as CAEBV‐B (blood) and CAEBV‐S (saliva)], a blood sample from a patient with primary immunodeficiency disorder (PID‐B), post‐transfusion lymphoproliferative disease (in blood, PTLD‐B, and in tumour, PTLD‐T), post‐solid organ transplant viraemia (in blood SOT‐B) and Hodgkin lymphomas (tumour, HL‐T) (Table SI).

### Statistical and sequence analysis

Samples were sequenced and analysed using a standard pipeline and in‐house R scripts (Supplementary Methods; Figure S1). The methods for diversity calculations and haplotype reconstruction have been described elsewhere.^8^, ^9^

## Results and discussion

### EBV genomes in blood and saliva from CAEBV patients

The EBV blood genomes from patients with CAEBV clustered with European/USA sequences; longitudinal samples, including those after PBSCT, clustered by patient (Figure S2). Within‐host CAEBV nucleotide diversity (p) was low and comparable to other EBV blood samples (Table SI, Figures S3 and S4). In contrast, CAEBV salivary samples showed significantly higher EBV diversity compared to blood and tumour samples (*P* < 0·001), indicating the presence of multiple strains (Figures S5–S7). Single nucleotide variants (SNVs) and small (<2 kbp) deletions were present in all specimens, including samples from healthy‐S and IM‐B. The SNVs and deletions were largely located in latent genes [e.g. Epstein–Barr nuclear antigen 3 (*EBNA3*), latent membrane protein 1/2 (*LMP1/2*)], EBV large tegument protein 1 (*BPLF1*) and EBV envelope glycoprotein GP350 (BLLF1) (Figures S8–S16). Our results showed EBV shed in saliva has the hallmarks of lytic replication and frequent mixed infections consistent with previous studies.^10^

### Larger deletions

We identified low frequency (<50% within‐host viral genomes) larger (>2 kb) EBV deletion in CAEBV patient 1 (one of four) (128 013–139 461 bp), as well as PTLD (two of four) and HL (two of seven) tumour‐tissue at position 120 470–158 062 (Fig 1B; Figure S17). This includes the *Bam‐*HI A rightward transcript (BART) microRNA (miRNA) clusters, several lytic genes, including scaffold proteins (e.g. BdRF1, BVRF2), glycoproteins (e.g. BXLF2), a tegument protein (BVRF1), and regulators of late gene transcription (BcRF1, BVLF1). Interestingly, patient 1 also had a nonsense variant in BXLF2 (>50% frequency, Fig 1C). A second larger deletion at positions 12 118–15 159, overlapping the major EBV repeat (IR1), was present in blood from CAEBV (four of four) and PID patients (Fig 1B). This deletion overlaps with a smaller deletion found in a PTLD tumour (one of four) at 11 494–12 322 bp.

### EBV variation overtime in patients with CAEBV

Analysis of variation over time revealed that the blood from CAEBV patient 1 with EBV present predominantly in T cells, showed higher genomic heterogeneity with increasing number of low frequency (<50%) variants and deletions compared to other patients (Fig 1D; Figures S18–S20). Deletions, the BXLF2 nonsense variant and other non‐synonymous SNVs overall persisted after rituximab treatment (Fig 1D). Clinical deterioration necessitated PBSCT (Table I), following which SNVs and deletions were absent from the virus reactivating asymptomatically.

**Table I:** Patients’ descriptions and clinical details.

Pat	Age, years	Sex	Description	Treatment	Transplant	Reactivation	*N* samples
P1	6	M	EBV‐driven HLH Primary and secondary immunodeficiency excluded	HLH 94 protocol 5 doses of Rituximab (pre‐transplant)	Alemtuzumab/fludarabine/melphalan conditioned MUD PBSCT	4 months post PBSCT had reactivation of EBV without signs of PTLD and resolved without therapy	7
P2	2·5	F	EBV‐driven HLH Primary and secondary immunodeficiency excluded	HLH 94 protocol 4 doses of rituximab	The patient did not proceed to BMT as she responded well to HLH treatment and had no evidence of ongoing HLH activity despite ongoing EBV viraemia	Remained EBV PCR positive for 3·5 years without symptoms	2
P3	14	F	EBV‐driven HLH, spontaneously resolved without treatment Primary and secondary immunodeficiency excluded Over the following 6 months she had two further EBV‐related HLH relapses treated with steroids. Although her HLH associated symptoms rapidly responded, her EBV viraemia continued to rise During the third HLH relapse treatment course patient had multiple complications (renal failure requiring haemofiltration, atrial fibrillation requiring DC cardioversion, adenovirus and CMV viraemias, invasive candida parapsilosis enteritis, facial palsy)	4 doses of rituximab with successfully depleted B cells but not reduced EBV viral load (pre‐transplant) After third HLH relapse was commenced on etoposide as per HLH 94 protocol with resolution of clinical symptoms and reduction in EBV viral load 1 dose of rituximab after transplant	Alemtuzumab/fludarabine/treosulfan conditioned MSD PBSCT	EBV reactivation that resolved after 1 dose of rituximab	5

BMT, bone marrow transplantation; CMV, cytomegalovirus; DC, direct current; EBV, Epstein–Barr virus; HLH, haemophagocytic lymphohistiocytosis; MSD, matched sibling donor; MUD, matched unrelated donor; PBSCT, peripheral blood stem cell transplant; PCR, polymerase chain reaction; PTLD, post‐transfusion lymphoproliferative disease.

In contrast, patient 2 had fewer non‐synonymous SNVs and deletions with a picture like the blood from patients with PTLD with evidence of clonality. The deletion in IR1 was lost after rituximab (Fig 1D).

The CAEBV patient 3, who also failed to respond to rituximab, had several SNVs and showed the deletion in IR1. The deletion was not immediately lost after PBSCT (27 days after), but it disappeared afterwards (62, 69 days after), even though viraemia was still present (Fig 1A).

## Discussion

The pathogenesis of CAEBV disease is not completely understood. In healthy people, EBV is latent in B cells, whereas in many patients’ clinical features of CAEBV are associated with the detection in T or NK lymphocytes, particularly in East Asia.^4^ It is unclear how EBV enters T and NK cells as they do not express the EBV complement receptor 2 (CR2 also known as CD21).

In the present study, we show that EBV deletions are present in all sequences, even from healthy individuals. These deletions, as well as SNVs, are found in latent genes and in a few lytic genes (e.g. BPLF1 and BLLF1), which are known to be under positive selection.^11^, ^12^

In addition, we found that blood, but not the saliva, of patients with CAEBV contained defective viral genomes, affecting BART miRNAs and late lytic genes (128 013–139 461 bp). Overlapping this region, we also identified one nonsense mutation in CAEBV samples affecting the glycoprotein H (BXLF2). Similar deletions are also found in malignancies, including tumours from PTLD and HL and extra‐nodal NK/T cells lymphoma and EBV^+^ diffuse large B‐cell lymphoma.^5^ Data from mouse models suggest that loss of the BART miRNAs may drive a more lytic phenotype and faster cell growth, predisposing to tumour formation.^7^, ^13^ Although, the mechanism is not entirely clear, the abortive EBV replication resulting from the absence of these regions may prevent normal cell death associated with lytic replication.^7^, ^14^ The cells remain exposed to continued high expression of lytic genes, driven by immediate early promotors, BZLF1 and BRLF1, which always remain intact. However, only one patient with CAEBV in this study (and 35% Japanese patients^5^) have deletions in this area and the others did not show any specific nonsense/missense variants, so further work is needed to determine the role of deletions in the CAEBV disease pathogenesis.

We also identified a 3k deletion at the 5′ of the EBV genome (12 118–15 159 bp) in samples from patients with CAEBV and PID. The deletion overlapped with the EBV internal repeat (IR1) region and will require further confirmation using long‐read sequencing. However, it overlaps with a smaller deletion (828 bp) found in the tumour of a patient with PTLD. This area includes BWRF1 and Wp, whereas the PTLD deletion affects the Cp. The identified deletions overlap with deletions found in Japanese CAEBV, diffuse large B cell and extra‐nodal NK/T cell lymphoma.^5^ Further tests *in vitro* showed that a deletion of Cp resulted in a higher rate of B‐cell growth transformation.^15^

Although similar EBV deletions have previously been described in CAEBV infection and malignancies,^5^, ^6^, ^7^ most were in patients of Asian origin. In the present study, we show that defective genomes occur independently of sample and patient geographical origin and are not necessarily associated with strains predominantly circulating in Asian countries or from patients of Asian origin.

Persistence of larger deletions in longitudinally sampled blood has been associated with clonal expansion of the cells in which they are found.^7^ Importantly, we show that clinical response to therapy is associated with loss of deletions and nonsense variants in virus recovered after treatment. The short‐term persistence following PBSCT of the deletion in patient 3’s EBV may reflect a longer half‐life of the lymphocyte clone in which it was located.

Our present findings of low‐level potentially premalignant clones together with evidence of replicating virus in blood from patients with CAEBV fits with the hypothesis that deletions occur only when a particular subset of the patient’s blood cells are infected with EBV. Progenitor lymphoid cells, which overtime differentiate into B, T, or NK cells, have been suggested to be the target cell type.^14^ Rituximab and/or PBSCT may remove the clone and when the same virus reactivates after treatment, we found it no longer contained the defective genomes.

In conclusion, our present study showed that clones containing EBV genomes with specific large deletions are found in blood but not saliva from European patients with CAEBV. While rituximab reduced the burden of EBV deletions in some cases, it had no impact on the prevalence of EBV deletions in T/NK‐cell associated CAEBV. The absence of these EBV‐deletions from virus reactivating following PBSCT suggests the loss of the clone. The possibility that these deletions could be useful biomarkers for monitoring the success of treatments for CAEBV now needs to be investigated in larger studies.

## Author contributions

Judith Breuer conceived the study, Cristina Venturini and Judith Breuer designed the study and wrote the manuscript; Cristina Venturini performed bioinformatics analysis with contributions from Sofia Morfopoulou and Fanny Wegner; Austen J. Worth, Anupama Rao and Persis J. Amrolia were responsible for care of the patients and provided clinical details. Charlotte J. Houldcroft, Arina Lazareva, Zainab Golwala collected clinical samples and data. Stephen D. Marks, Jacob Simmonds, Jeffrey I. Cohen, Tetsushi Yoshikawa and Paul J. Farrell provided additional samples and data. All authors read and participate in critical revision of the article.

## Conflict of interest

The authors declare no conflict of interest.

## Supporting information


**Data S1**.
**Fig S1**. Boxplot showing the average depth, excluding duplicate reads and repeated regions, for each sample coloured by clinical group.
**Fig S2**. MDS clustering of all samples coloured by patient group. Multiple samples from chronic active Epstein–Barr virus (CAEBV) patients P1, P2, P3 and the paired blood‐saliva (P4) are indicated.
**Fig S3**. The *y*‐axis shows the within host nucleotide diversity (π) per sample by group (*x*‐axis and colours). Vertical grey dashed line shows lower and upper quartile, and the grey dot is the median.
**Fig S4**. Relationship between average depth (*x*‐axis) and diversity calculation (*y*‐axis). Higher diversity is not associated with higher depth (minimum depth shown is ×10; see also Table SI).
**Fig S5**. Frequency distribution of variants (obtained by mapping against their own consensus) for (A) blood SOT samples suspected as harbouring mixed infections (B) all salivary chronic active Epstein–Barr virus (CAEBV) samples and (C) saliva from an asymptomatic shedder.
**Fig S6**. Haplotype frequency after haplotype reconstruction (HaROLD)^8,9^ in SOT blood samples (A), saliva samples from patients with chronic active Epstein–Barr virus (CAEBV) (B) and asymptomatic shedder (C). The majority haplotype is coloured pink by convention with additional haplotypes coloured green and yellow.
**Fig S7**. Multidimensional scaling (MDS) clustering including all EBV sequences included in the paper (in grey) and the reconstructed haplotypes sequences.
**Fig S8**. Relationship between number of SNVs in total (including all types of coding and non‐coding mutations with at least 2% frequency and strict QC as explained in the ‘Methods’ section) and the average depth by sample coloured by disease group (minimum average depth is ×10).
**Fig S9**. Boxplots for number of single nucleotide variants (SNVs) in each EBV‐related disease/group. Significance: *P ≤ 0·05, **P ≤ 0·01.
**Fig S10**. Location of single nucleotide variants (SNVs), excluding samples with possible mixed infections in the EBV genome.
**Fig S11**. The most common non‐synonymous mutations are coloured by disease group.
**Fig S12**. Total number of non‐synonymous single nucleotide variants (SNVs) by sample (only samples with non‐synonymous variants are shown) and genes involved by category. The size of the filled circles represents the number of variants.
**Fig S13**. Size of deletions in all samples, coloured by group. First plot (at the top) shows the full range of deletion’ sizes (1–24 474). The second plot is a zoom for deletions with sizes between 1 and 100. Indels with a size <30 bp were present in all groups and considered artefacts.
**Fig S14**. Distribution of size of deletions (left, all deletions; right, a zoom of sizes from 30 to 10 kbp). Most (95%) deletions were smaller than 2 kbp, therefore that was chosen as cut‐off to distinguish ‘small’ deletions *versus* ‘big’ deletions.
**Fig S15**. Number of triplets (*x*‐axis) *versus* number of non‐triplets (*y*‐axis) by gene and coloured by group.
**Fig S16**. Total number of deletions (≥30 bp and <2 kbp in size) per sample coloured by clinical group (A) and the genes affected by deletions (B).
**Fig S17**. Genes affected by larger deletions (>2 kbp) in each sample coloured by clinical group.
**Fig S18**. Analysis of single nucleotide variants (SNVs) in longitudinal samples in patients with chronic active Epstein–Barr virus (CAEBV). Samples from three patients are shown in different colours. The top panel shows the number of all SNVs for each sample. Genes affected by non‐synonymous variants.
**Fig S19**. (A) Number of total single nucleotide variants (SNVs) and genes affected by non‐synonymous mutations for longitudinal blood samples for patients with post‐transfusion lymphoproliferative disease (PTLD) and infectious mononucleosis (IM). (B) Total number of bigger deletions (>2 kbp) and genes affected by deletions in longitudinal blood samples for IM. Patients with PTLD did not have any bigger deletions.
**Fig S20**. Summary of total number of deletions (A) and their position in the EBV genome (B) for chronic active Epstein–Barr virus (CAEBV) patient 4 in whole blood and saliva.
**Fig S21**. Summary of total number of deletions (A) and their position in the EBV genome (B) for the PID‐B patient in whole blood, T cells and saliva.
**Fig S22**. Single nucleotide variants (SNVs) in whole blood and T cells in the patient with primary immunodeficiency disorder‐blood (PID‐B). The *y*‐axis shows the position in the EBV genome (NC_007605.1) and the colour the type of mutation. The transparency indicates the frequency.
**Fig S23**. Representation of the IR1 deletion in chronic active Epstein–Barr virus (CAEBV). Representative portion of the alignment file (bam file) for a blood CAEBV sample to demonstrate IR1 deletion (represented by red reads and black lines) compared to an infectious mononucleosis (IM) blood sample where the deletion is not present (reads in grey).

**Table SI**. Details for each sample.

## Data Availability

Sequence reads for with CAEBV and PID have been deposited in the European Nucleotide Archive (ENA) under BioProject ID PRJEB41945. All accession numbers for the rest of the dataset are available in Table SI.
